# A Bioinspired Astrocyte-Derived Coating Promotes the In Vitro Proliferation of Human Neural Stem Cells While Maintaining Their Stemness

**DOI:** 10.3390/biomimetics8080589

**Published:** 2023-12-04

**Authors:** Andrea C. Jimenez-Vergara, Jacob Avina, Travis Jackson Block, Anne Sheldrake, Carson Koch, Anna Gonzalez, Jennifer Steele, Ana M. Díaz-Lasprilla, Dany J. Munoz-Pinto

**Affiliations:** 1 Engineering Science Department, Trinity University, San Antonio, TX 78212, USA; ajimene1@trinity.edu (A.C.J.-V.); jacob.avina2@gmail.com (J.A.); annaisabelglez@gmail.com (A.G.); adiazlas@trinity.edu (A.M.D.-L.); 2 StemBioSys, San Antonio, TX 78229, USA; travis.block@stembiosys.com (T.J.B.); anne.sheldrake@stembiosys.com (A.S.); 3 Neuroscience Program, Trinity University, San Antonio, TX 78212, USA; carsonkotx@gmail.com; 4 Physics and Astronomy Department, Trinity University, San Antonio, TX 78212, USA; jsteele@trinity.edu

**Keywords:** human neural stem cells, astrocyte-based coatings, proliferation, stemness

## Abstract

The repair of neuronal tissue is a challenging process due to the limited proliferative capacity of neurons. Neural stem cells (NSCs) can aid in the regeneration process of neural tissue due to their high proliferation potential and capacity to differentiate into neurons. The therapeutic potential of these cells can only be achieved if sufficient cells are obtained without losing their differentiation potential. Toward this end, an astrocyte-derived coating (HAc) was evaluated as a promising substrate to promote the proliferation of NSCs. Mass spectroscopy and scanning electron microscopy were used to characterize the HAc. The proliferation rate and the expression of stemness and differentiation markers in NSCs cultured on the HAc were evaluated and compared to the responses of these cells to commonly used coating materials including Poly-L-Ornithine (PLO), and a Human Induced Pluripotent Stem Cell (HiPSC)-based coating. The use of the HAc promotes the in vitro cell growth of NSCs. The expression of the stemness markers Sox2 and Nestin, and the differentiation marker DCX in the HAc group was akin to the expression of these markers in the controls. In summary, HAc supported the proliferation of NSCs while maintaining their stemness and neural differentiation potential.

## 1. Introduction

The average life expectancy of humans has increased dramatically throughout the world. Prior to the industrial revolution, in which the world saw massive leaps in technology, human life expectancy was around 42 years old. Today, children born into the world can expect to live about 80 years. This significant increase in human life expectancy as a result of various technological improvements and societal infrastructure has led the world to face a series of challenges people have never historically faced before. As people tend to reach older ages, the healthcare industry is tasked with combating the various neurodegenerative diseases and traumatic neurological injuries commonly developed in the older portion of the population. Some of the most common neurodegenerative disorders facing the elderly within society include Parkinson’s disease (PD), Alzheimer’s disease (AD), multiple sclerosis (MS), as well as cerebrovascular events such as stroke. These conditions are treated using various medications and therapies; however, there is no concrete treatment to completely halt the progression of these diseases or significantly reverse tissue damage within the nervous system. Therefore, strong efforts have been expended in recent years toward the development of new and effective strategies for the treatment of neurological disorders and neurological trauma. However, one particular and difficult challenge neurological treatments face is the limited capacity of neuron cells to proliferate while reestablishing synaptic connections [1,2].

In recent years, one critical route of research scientists have pursued to address this challenge has been focused on the use of induced pluripotent stem cells (iPSCs) to establish patient-specific neural stem cells (NSCs) which can be later used in cell-based therapies, transplants [3,4], disease modeling [5,6,7], and screening of drugs [8]. Reprogrammed from somatic cells, iPSCs are engineered pluripotent cells that behave similarly to embryonic stem cells, are capable of self-renewal, and have the potential to be differentiated into cells from the three germ layers including neuron cells [7]. Due to the limited availability of primary human neurons, their negligible proliferation capacity, and the ethical concerns regarding the use of embryonic stem cells, iPSCs have emerged as a preferential source of pluripotent cells to obtain NSCs. Under the right cell culture conditions, NSCs can be specifically differentiated into neuron-like cells. In addition, patient-specific human iPSC-derived NSCs (i-HNSCs) can be obtained, which limits the in vivo immune response and can facilitate the implementation of precision medicine. Despite the potential of i-HNSC, the use of these cells on an industrial and therapeutic scale is still restricted in part due to the limitations of current in vitro culture technologies.

One of the challenges for the in vitro culture and expansion of i-HNSCs is the limited ability of these cells to attach to traditional cell culture materials including cell culture-treated polystyrene (TCPS) and glass-based surfaces [9]. To enhance the attachment and promote the proliferation of these cells, culture surfaces are commonly coated with different types of substrates including small molecules, single proteins, a mixture of proteins, or extracellular matrix components (ECM). Single proteins such as laminin, collagen type I, and fibronectin; small molecules including poly-l-ornithine (PLO); and integrated materials such as Matrigel or solubilized iPSC ECM components have been traditionally used to promote the proliferation and differentiation of i-HNSCs [10,11]. These cells generally have a positive response to these types of coatings and exhibit better attachment and proliferation relative to uncoated surfaces. However, small-molecule- and single-protein-based coatings dramatically oversimplify the complexity of the microenvironment that NSCs experience in vivo. The neural stem cell niche plays an important role in regulating cell–cell interactions, the architecture of the microenvironment, and the spatiotemporal modulation of biochemical cues such as fibroblast growth factor-2 (FGF-2), vascular endothelial growth factor (VEGF), and transforming growth factor beta-2 (TGFβ-2) [12,13,14]. Complex protein mixtures such as Matrigel and soluble iPSC ECM molecules attempt to mimic in a more comprehensive manner the biochemical landscape of the cellular niche of NSCs in nervous tissue but cannot fully replicate the architecture and complexity of the cellular milieu present in neural tissue. To address these limitations, our team hypothesized that the use of an astrocyte-derived matrix as a bioinspired material may be more realistic and could be a better substrate than traditional coatings at mimicking the main key microenvironmental features that NSCs may experience in vivo. Astrocytes are the most abundant glial cells in central nervous system (CNS) tissue. These cells were selected in this study as the ECM source for our proposed bioinspired coating because in vivo astrocytes are primarily responsible for ECM deposition and the promotion of CNS tissue homeostasis [15,16]. Towards this end, a confluent monolayer of primary human astrocytes was decellularized by the use of hypertonic-based solutions to produce a substrate that retains the biochemical (interconnected fibrillar network of ECM proteins, polysaccharides, and other macromolecules), topological, and biomechanical cues of the cell monolayer [17,18]. Decellularized matrices have been successfully used in other applications to improve the cellular responses of human chondrocytes, human adult stem cells, and cardiomyocytes [19,20,21]. In addition, this substrate fabrication approach has also been used for the proliferation and differentiation of human iPSCs. Cho et al. used decellularized human brain tissue to generate functional oligodendrocytes from human iPSCs [22]. Carvalho et al. developed a decellularized cell-derived ECM from neural progenitor cells which was able to promote the growth of human iPSCs without altering their pluripotency potential and with the capability to enhance the differentiation toward neuron-like cells [10]. Although progress has been made in this type of substrate, there is still a need for the development of new decellularized ECM substrates for the expansion of human iPSC-derived NSCs.

**Figure 1:**
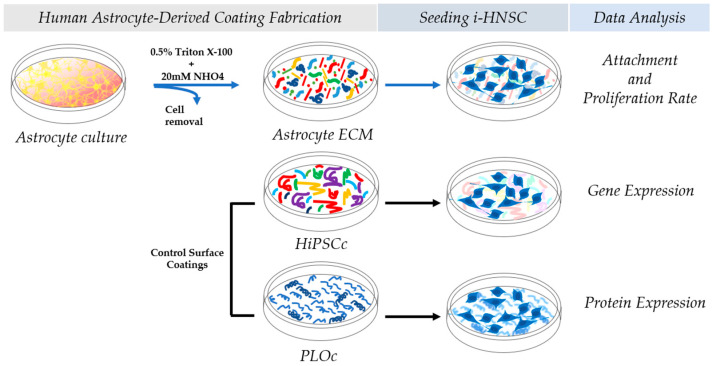
Schematic representation of the experimental design for the fabrication of a HAc and its initial characterization.

In this exploratory work, we successfully fabricated a human astrocyte-based coating (HAc). The substrates were characterized using microscope phase contrast images, scanning electron microscopy, and mass spectroscopy. The biological responses of i-HNSCs grown in growth media to HAc culture surfaces were characterized in terms of the proliferation rates (specific growth rate and doubling time), the expression of the stemness markers Sox2 and Nestin, and the neural differentiation marker DXC. Surfaces coated with PLO and iPSC ECM were used as our control groups. The capacity of the i-HNSCs to differentiate into neuron-like cells on the HAc was evaluated after seven days in culture using neural differentiation media. The expression of the stemness marker Sox2 and the neuron markers NeuroD1, RBFox3, and MAP2 were evaluated at the gene expression level. A schematic summary of the experimental design is shown in Figure 1. In summary, our results indicate that i-HNSCs cultured on HAc exhibited similar or higher proliferation rates than in the control surfaces while maintaining consistent levels of expression of stemness markers when cultured in growth media. Finally, the expression of neural markers was enhanced in i-HNSCs cultured using neuron differentiation media, demonstrating that i-HNSCs cultured on the HAc retained their neural differentiation potential.

## 2. Materials and Methods

### 2.1. Human Astrocyte-Derived Coating Fabrication

The astrocyte-derived ECM was produced under aseptic conditions using procedures adapted from previously reported research work by Marinkovic et al. for other cell types [23]. Primary human astrocytes from the cerebral cortex (ScienCell Research Laboratories, Carlsbad, CA, USA) were seeded on fibronectin-coated culture dishes and cultured to confluence in Astrocyte Medium (ScienCell) with media changes every 3–4 days. When the cells reached confluence, the cell culture medium was supplemented with 60 mg/L ascorbic acid (Sigma-Aldrich, Inc., St Louis, MO, USA) to induce matrix protein secretion. The resulting matrix was washed with phosphate-buffered saline (PBS) and then decellularized for 7 min at room temperature with PBS containing 0.5% Triton X-100 and 20 mM NHO_4_. The decellularized ECM was then washed twice with PBS and once with sterile distilled water, and then allowed to air dry for long-term storage at 4 °C. Immediately prior to use, the dried and decellularized matrix was rehydrated by incubation with PBS. The described ECM is now commercially available under the product name CELLvo NeuroMatrix (StemBioSys Inc., San Antonio, TX, USA).

### 2.2. Mass Spectroscopy

The protein composition of the extracellular matrix (ECM) was determined using previously described procedures [23]. ECM proteins were removed from cell culture dishes using a rubber policeman and solubilized. Protein separation was performed using a one-dimension SDS-PAGE and Coomassie blue staining. Bands of interest were excised and proteins were digested with trypsin. The digests were analyzed using capillary HPLC–electrospray ionization tandem mass spectrometry on a Thermo Fisher LTQ fitted with a New Objective PicoView 550 nanospray interface. Protein and peptide identity probabilities were determined using Scaffold (Proteome Software Inc., version: 5.3.0). Protein identifications were accepted using the following criteria: minimum number of peptides, 4; peptide probability, ≥99%; protein probability, ≥99%.

### 2.3. Control Surface Coatings

Polystyrene well plates were coated using Poly-L-Ornithine (PLO, Sigma–Aldrich) and Human Induced Pluripotent Stem Cell (HiPSC) coating solution (Cell Applications, Inc., San Diego, CA, USA). Well plates were coated with 0.26 mL/cm^2^ of 20 µg/mL PLO solution in DPBS. The HiPSC coating solution was used according to the protocols and recommendations of the manufacturer (Cell Applications).

### 2.4. Microscopic Characterization

The deposition of the human astrocyte-based ECM on TCPS was qualitatively characterized using phase contrast microscopy. Images were collected following the decellularization step using an Olympus XC50 microscope. In addition, scanning electron microscopy (SEM) images were obtained of the HAc and the control surfaces using a JEOL-JSM-6010LA scanning electron microscope (JEOL, Pleasanton, CA, USA). In brief, the three coating surfaces were fixed with 100% *w*/*w* ethanol for 60 min, treated with hexamethyldisilazane (Polysciences Inc., Warrington, PA, USA), and allowed to air dry for 24 h. Before imaging, the samples were coated with gold to a thickness of approximately 5 nm using a Cressington 208 auto sputter coater. At least 4 images were taken from random locations in each sample specimen at 5.0 kV and 7000× magnification.

### 2.5. Cell Culture

Cryopreserved Human iPSC-Derived Neural Stem Cells (i-HNSCs, Cell Applications, Inc.) were thawed and expanded. i-HNSCs were cultured in growth media: DMEM (Dulbecco’s Modified Eagle’s Medium)/F12 50:50 (Corning, Manassas, VA, USA) supplemented with 2% B-27 Plus Supplement (50X, Gibco, Carlsbad, CA, USA), 2 µg/mL heparin (Fisher, Waltham, MA, USA) + 0.02 µg/mL bFGF (basic fibroblast growth factor, PeproTech, Cranbury, NJ, USA) + 0.02 µg/mL EGF (epidermal growth factor, PeproTech, Cranbury, NJ, USA) + 1% Glutamax (Gibco) + 1% PS (penicillin–streptomycin, Gibco). Cells were expanded at 37 °C, 5% CO_2_, and oxygen levels estimated to be 18.6 O_2_% *v*/*v* [24]. Cells were collected between passages 4 and 5. Then, i-HNSCs were seeded onto the coated surfaces at approximately 15,000 cells/cm^2^ using growth media +0.5X rho-associated protein kinase (ROCK) inhibitor. The ROCK inhibitor was used to increase cell survival. The next day, the medium was changed to growth medium (GM) and the seeded i-HNSCs were cultured in this medium for 96 h. Samples were collected for cell attachment 24 h after seeding, cell proliferation samples were collected 24 h, 48 h, and 96 h after GM exposure, and gene expression and protein analysis were evaluated 24 h after seeding.

To evaluate the retention of the neural differentiation potential of i-HNSCs on our HAc, cells were seeded at approximately 40,000 cells/cm^2^ on a 6-well plate. Following the 24 h treatment with the ROCK inhibitor, samples of the undifferentiated cell population were collected (control, n = 3 wells). The remaining cells were cultured in Neural Differentiation Media (NDM, Cell Applications) for seven days at 37 °C, 5% CO_2_, and oxygen levels estimated to be 18.6 O_2_% *v*/*v* (n = 3 wells). Samples from the 0-day (control) and the 7-day groups were collected for gene expression analysis.

### 2.6. Attachment and Proliferation Rate Analysis

To assess i-HNSC attachment and proliferation on the different surfaces, brightfield images of attached cells were taken at 24 h after seeding using a Nikon Eclipse TS100 (Nikon, Melville, NY, USA) microscope equipped with a DS-Ri1 Nikon microscope camera. After cell attachment was verified, samples from each type of coating were collected to evaluate the proliferation of the cells after 24 h, 48 h, and 96 h in GM. In brief, well surfaces were rinsed once with DPBS and then lysis buffer from the Dynabeads mRNA direct kit (Ambion, Life Technologies, Carlsbad, CA, USA) was added. Samples were incubated for 10 min at room temperature, collected, and stored at −80 °C until further use. Then, DNA levels were used to calculate cell numbers, the specific growth rate, and the doubling time for each population of cells. DNA was quantified from the collected samples using the PicoGreen dsDNA quantitation assay (Invitrogen, Life Technologies, Carlsbad, CA, USA). DNA levels were converted to cell numbers using 6.6 pg DNA per cell as the conversion factor, and cell density per area was calculated as the number of cells per area. The proliferation profiles for each treatment were evaluated by calculating the characteristic specific growth rate and the doubling time. Each proliferation profile was fit with an exponential curve, and the specific growth rate was calculated from the exponential model and used to determine the doubling time (*t_D_* [h]) as shown in Equations (1) and (2). *X* [cells/cm^2^] is the cell population at any given time point, *X*_0_ is the extrapolated initial cell population [cells/cm^2^], and µ is the specific growth rate [h^−1^].

(1) $$X=X_{0}e^{µt},$$

(2) $$t_{D}=\frac{\ln(2)}{µ},$$

### 2.7. Gene Expression Characterization of Stemness Markers

After 24 h, messenger RNA (mRNA) was extracted using the Dynabeads mRNA direct kit (Ambion, Life Technologies). In brief, surfaces were rinsed with DPBS and then lysis buffer was added. Samples were incubated with the lysis buffer for 10 min of incubation and then collected and stored at −80 °C until further use. After thawing, the polyA-mRNA was extracted using 20 µL of Dynabeads oligo (dT)25 magnetic beads. Then, the resulting mRNA-beads were washed twice with buffers A and B. mRNA-beads were re-suspended in ice-cold 10 mM Tris–HCl, followed by the mRNA elution from the beads by heating the solutions at 80 °C for 2 min. The supernatant was collected and stored at −80 °C until further use [25].

The relative gene expression of i-HNSCs was calculated using a 7500 Real-Time PCR System (Applied Biosystems, Waltham, MA, USA) and the SuperScript III Platinum One-Step qRT-PCR kit (Invitrogen, Life Technologies). mRNA levels for the neural stem cell genes SRY (sex determining region Y)-box 2 (Sox2) and Neuroepithelial Stem Cell Protein (Nestin) and for the immature neuron marker Doublecortin (DCX) were analyzed. Primer sequences are shown in Table 1. A reaction mixture of 25 µL was used with approximately 6 ng of polyA-mRNA and 5 µL of 1 mM primer. Changes in SYBR Green fluorescence were used to monitor the reaction amplification and ROX dye was used as the passive reference. The ΔΔCt method was used to calculate the gene expression for each sample and beta-actin (β-actin) was selected as the housekeeping gene. Melting temperatures were used to verify the appropriate amplification products for each PCR reaction.

### 2.8. Protein Expression Assessments of Stemness Markers

Protein expression was evaluated by immunostaining. After 24 h in culture, samples from each coating surface were washed with DPBS once, fixed with formalin, and stored at 4 °C until use. The expression of the neural stem cell markers (Table 2) Sox2 and Nestin was assessed. In brief, wells were rinsed with PBS and blocked with 3% BSA diluted in PBST (PBS + 0.05% Tween 20) for 1 h. Primary antibody diluted in a 3% BSA–PBST solution was added and incubated for 1 h at room temperature. Following rinsing with PBS, a 3% BSA–PBST solution containing Alexa 488 anti-mouse antibody (Life Technologies) and DAPI dilactate (4′,6-diamidino-2-phenylindole, Life technologies, 300 nM) was added to each sample and incubated for 1 h. The samples were rinsed with PBS three times and images of the immunostaining were taken using a Nikon A1 confocal microscope system equipped with a 10X objective. A total of 12 pictures were taken from 3 independent specimens per experimental group.

Semi-quantitative evaluation of the immunostaining results was used to assess the protein expression of the selected markers. The number of cells stained with DAPI (total cell number) and the number of cells stained with Alexa 488 anti-mouse antibody (number of cells stained) was determined by cell counting. The cell counts were assessed using the multi-point tool in ImageJ software. The fluorescence intensity for each Alexa 488-stained image was quantified using Photoshop. The overall immunostaining intensity (*d*) for each image was found using Equation (3). The average and standard deviation were calculated for the overall intensity for each experimental group. The relative expression of protein expression was calculated using the HiPSCs as the reference group.

(3) $$d=\frac{Number\;of\;cells\;stained\times Fluorescence\;intensity}{Total\;cell\;number}$$

### 2.9. Gene Expression Characterization of Neural Differentiation Markers

The differentiation potential of i-HNSCs on the HAc was evaluated after 7 days in culture using NDM. Undifferentiated cells (0-day group) were used as our control population. mRNA was extracted and qRT-PCR measurements were performed following the described methods summarized in Section 2.7. The expression of the stemness marker Sox2, the immature neuron marker Neurogenic Differentiation 1 (NeuroD1), and the mature neuron markers RNA-Binding Protein Fox-1 Homolog 3 (RBFox3) and Microtubule-Associated Protein 2 (MAP2) was assessed using the primer sequences reported in Table 1 [26,27].

### 2.10. Statistical Analyses

All quantitative data results are reported as the mean ± standard deviation. Comparison of arithmetic average across samples was performed using ANOVA followed by Tukey’s post hoc test (IBM SPSS Statistics software, version: 29.0.1.0). Significant differences among experimental groups were considered for *p* < 0.05.

## 3. Results

### 3.1. Mass Spectroscopy

Mass spectrometry data were collected from ECM produced by three astrocyte donors and 280 proteins were identified, with 145 being found in all three. Intracellular and intranuclear proteins were among those identified. Their presence results from the lysis of the astrocytes used for ECM production. When cross-referenced against Matrisome DB 2.0, a total of 44 extracellular matrix proteins were identified, with 29 proteins identified in all three samples. Differences in extracellular matrix composition between the three donors were limited to half of the proteins with the lowest abundance.

**Figure 2:**
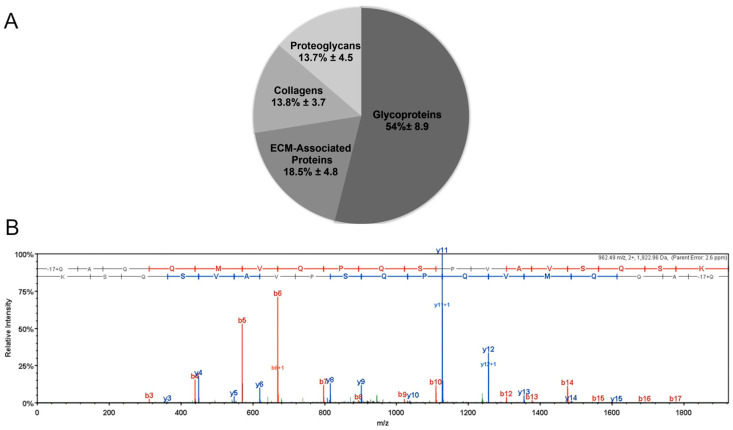
The composition of astrocyte-derived ECM was broken down by type of matrix component. Glycoproteins made up the largest portion, followed by ECM-associated components, collagens, and proteoglycans (**A**). Representative mass spectrum of fibronectin for one of the sample donors. The fragments identified to extend from the carboxyl terminus (y ions) are in blue and those identified to extend from the amino terminus (b ions) are in red (**B**).

Across all donors, the most abundant ECM components included fibronectin, heparan sulfate, tenascin, TGFB1, and serpin H1. The most common collagens identified were collagen I, collagen VI, collagen II, and collagen VIII. By spectral counts, approximately 13.8% of the ECM was composed of collagens. A representative and example mass spectrum showing the relative abundance vs. *m*/*z* for fibronectin from the astrocyte matrix sample for one of the donors is shown in Figure 2.

### 3.2. Microscopic Characterization

**Figure 3:**
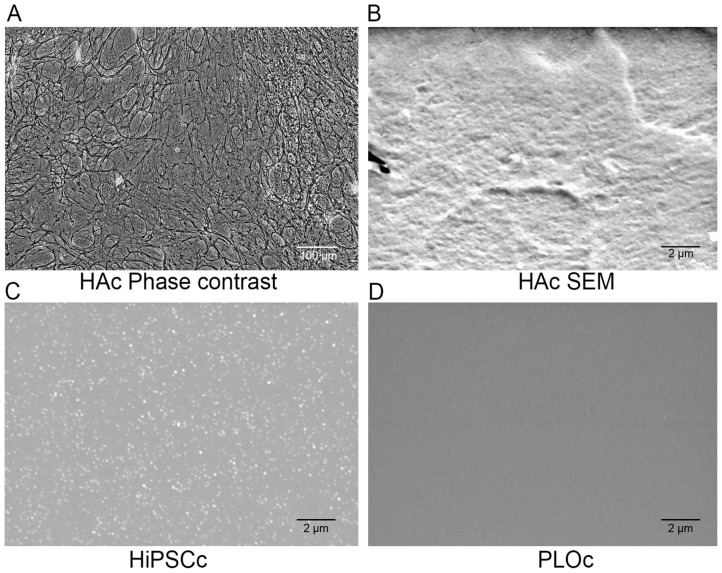
Representative phase contrast image of the decellularized astrocyte monolayer; scale bar = 100 µm (**A**). Representative SEM image of HAc; scale bar = 2 µm (**B**). Representative SEM image of HiPSCc; scale bar = 2 µm (**C**). Representative SEM image of PLOc; scale bar = 2 µm (**D**).

Phase contrast microscopy was used to confirm the successful decellularization process of the astrocyte monolayer (Figure 3A). Cellular structures such as cell nuclei are not present in the image, while a network of ECM fabric is clearly visible. In addition to the phase contrast image characterization, SEM was used to evaluate differences at the microscale for the different coated surfaces. As expected, qualitative information from the SEM data reveals a dense, packed, and moderately smooth surface in the HAc (Figure 3B). In contrast, the HiPSCc shows a discrete set of macromolecule clusters evenly distributed across the landscape. The clusters of macromolecules appear to be between 100 and 150 nm in size. The PLO-coated surface (Figure 3C) displays a very smooth surface with no distinguishable structures at the micro- or nano-scale. Differences in the surface of the coating may be due to differences in the total concentration of macromolecules in the coating and the size of the molecular components.

### 3.3. Attachment and Proliferation of i-HNSCs

**Figure 4:**
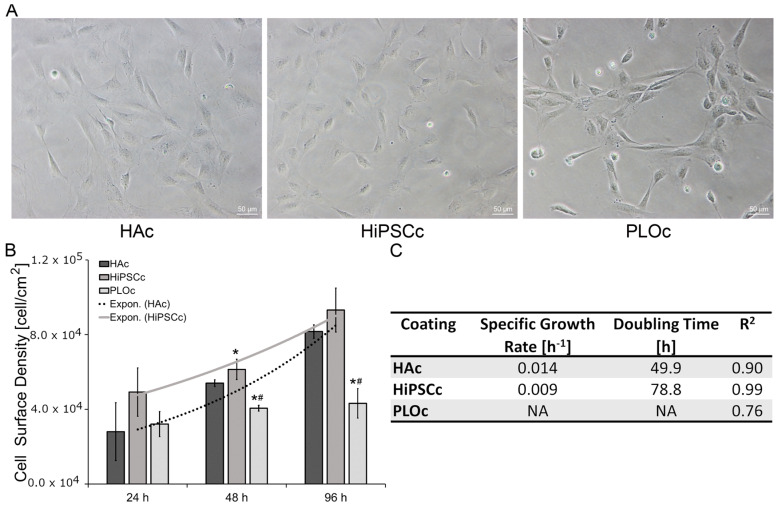
Representative brightfield images of i-HNSCs attached to the different coatings 24 h after seeding; scale bar = 50 µm (**A**). Cell proliferation profiles of i-HNSCs cultured in the different coated surfaces (**B**). Specific growth rate and doubling time (**C**). * Significantly different from HAc; # significantly different from HiPSCc. *p* < 0.05; n = 4.

i-HNSCs were seeded on surfaces coated with HAc, HiPSCc, and PLOc to evaluate the potential of the HAc surface to support i-HNSC adhesion and proliferation. As shown in Figure 4A, i-HNSCs attached to all three coated surfaces. However, cells in the HAc and HiPSCc surfaces appeared to be evenly distributed over the whole surface, while in the PLOc, the cells attached to localized regions of the surface. Following the initial evaluation of attachment, cells were cultured in growth media and samples were collected at 24 h, 48 h, and 96 h. At these time points, cells were lysed and cell surface density was calculated using DNA measurements (Figure 4B). At 24 h, cell surface density results indicated a lower initial attachment to HAc relative to the other two coatings. Cell surface density on the HAc was 1.8- and 1.1-fold lower than the density on the HiPSCc and PLOc, respectively. Similarly, cell surface density was 1.5-fold lower in the PLOc formulation relative to HiPSCc. However, no significant differences were found at 24 h (*p* ≥ 0.086). To assess cell proliferation, samples were also collected at 48 h and 96 h. As it is shown in Figure 4B, at 48 h, the cell surface density was 1.1-fold lower in the HAc surface relative to HiPSCc (*p* = 0.035). However, the cell surface density was 1.3-fold higher in the HAc formulation relative to PLOc (*p* = 0.001). Similarly, the cell surface density was 1.5-fold higher in the HiPSCc surface relative to PLOc (*p* = 0.000). At 96 h, the results showed similar trends (Figure 4B). The cell surface density was 1.1- and 2.2-fold higher in the HiPSCc sample relative to HAc and PLOc, respectively. However, the number of cells per area in the HiPSCc was only significantly different from PLOc (*p* ≤ 0.001). Similarly, cell surface density was 1.9-fold higher in the HAc sample relative to PLOc (*p* ≤ 0.001). Using the surface cell density, the specific growth rate and the doubling time for the i-HNSCs were calculated for the HAc and HiPSCc coating (Figure 4C). Cells on the PLOc proliferated at a very low rate and did not reach the exponential growth phase during the time frame of the experiments. The estimated doubling time of i-HNSCs on the HAc was 49.9 h and 78.8 h on the HiPSCc. While these values seem different, they are rough estimates given the very limited number of data points for the exponential model and the difference in value may be a mathematical artifact.

### 3.4. Relative Gene and Protein Expression Results

**Figure 5:**
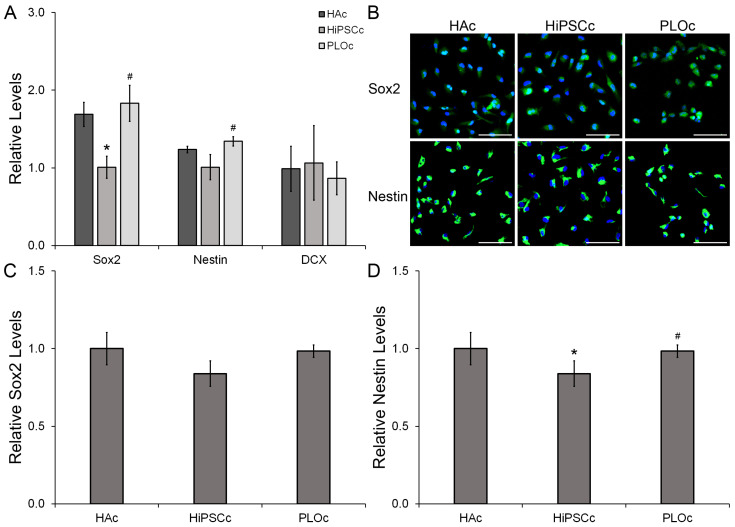
Phenotypical evaluation of i-HNSCs 24 h after seeding using qRT-PCR, n = 4 (**A**). Representative immunofluorescent images in green color for the Sox2 and Nestin stainings with DAPI as counterstaining in blue (**B**). Semi-quantitative analysis of immunostaining results for the protein expression of Sox2 (**C**) and Nestin (**D**) 24 h after seeding. * Significantly different from HAc; # significantly different from HiPSCc. *p* < 0.05; n = 3. Scale bar = 100 µm.

To evaluate the initial effects of HAc on i-HNSC behavior, samples were collected 24 h after seeding. Gene expression results (Figure 5A) showed a 1.7-fold decrease in the neural stem cell marker Sox2 for the PLOc surface relative to HAc (*p* = 0.009). Similarly, the levels of Sox2 were 1.8-fold lower in the PLOc formulation relative to HiPSCc (*p* = 0.003). The expression of Sox2 in the HiPSCc sample was slightly increased relative to HAc; however, there were no significant differences between these two groups (*p* = 0.626). In the case of the neural stemness marker Nestin, the relative expression of this marker was decreased in the HiPSCc relative to HAc and PLOc; however, significant differences were only found in the PLOc group (*p* = 0.017). Nestin was downregulated 1.2- and 1.3-fold relative to HAc and PLOc, respectively. In addition, there were no significant differences in Nestin levels between HAc and PLOc. The gene expression of the neural immature marker DCX was also evaluated. The relative levels of DCX were similar in the three coatings used in this study.

In terms of protein expression (Figure 5B–D), Sox2 was identified in both the nucleus and the cytoplasm (Supplementary Figure S1). Although Sox2 is a transcription factor, its acetylated form is transported into the cytoplasm [28,29]. The relative levels of Sox2 were reduced 1.2-fold in the HiPSCc formulation relative to the other two coatings. However, no significant differences were found between the groups (*p* ≥ 0.054). In addition, Sox2 levels were similar between the HAc and the PLOc group. Similarly, the relative levels of Nestin between HAc and PLOc were statistically indistinguishable. However, the levels of this marker were 1.1- and 1.2-fold lower in the HiPSCc surface relative to HAc (*p* = 0.003) and PLOc (*p* ≤ 0.001), respectively.

**Figure 6:**
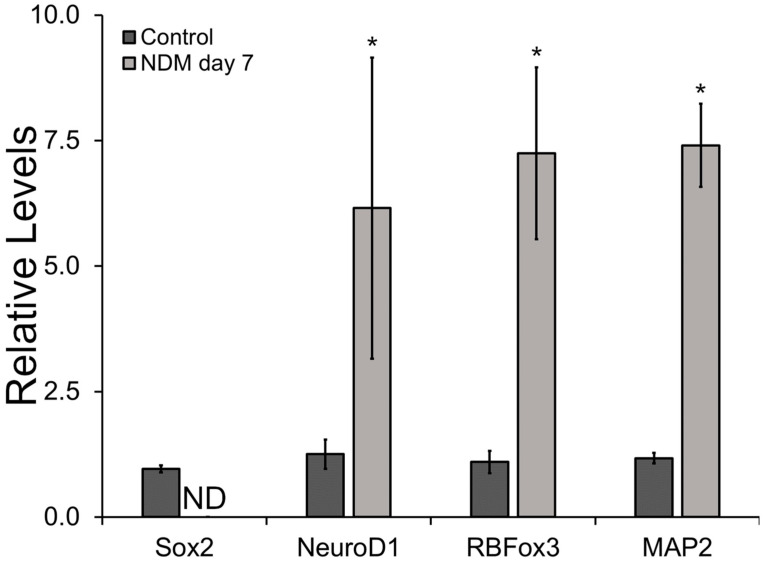
Phenotypical evaluation of i-HNSCs cultured in NDM after 7 days in culture using qRT-PCR. * Significantly different from the 0-day control group; n = 3. ND = Below the detection limit.

To assess the neural differentiation potential of i-NSCs on the HAc, the expression of the stemness marker Sox2 and the neural differentiation markers NeuroD1, RBFox3, and MAP2 was evaluated at the gene expression level following 7 days in NDM (Figure 6). The expression of Sox2 decreased significantly after 7 days in culture. The expression of this marker was below the detection limit of the technique under reported experimental conditions. In contrast, the expression of immature neural marker NeuroD1 increased 6.2-fold after seven days in culture relative to the control group (*p* = 0.048). Similarly, the expression of neural mature markers RBFox3 and MAP2 after seven days in NDM was upregulated 7.2- (*p* = 0.04) and 7.4-fold (*p* ≤ 0.001), respectively, relative to the undifferentiated cells.

## 4. Discussion

One current challenge for the treatment of neurological diseases and neural trauma is the limited proliferative capacity of neurons. Endogenous NSCs are normally the preferred source of cells since these cells do not trigger an immune response. However, endogenous cells are not always available, particularly in elderly patients or ill individuals exhibiting chronic conditions. Exogenous NSCs are a potential alternative, but this potential source of cells may induce adverse immunological responses in the host, limiting neurogenesis [30,31,32]. To address this limitation, neural stem cells have been identified as a suitable cell source of neurons and the development of new therapies and treatments. These cells can be obtained from patient-specific induced pluripotent stem cells [33]. The success of the use of stem cells relies on the capacity of these cells to yield therapeutic numbers while maintaining their stemness and differentiation potential. To accomplish this goal, research efforts have been focused on recapitulating and mimicking key elements of the stem cell niche [14]. The NSC niche is characterized by the presence of various cell types such as endothelial, pericytes, astrocytes, and microglia [13]. In this work, we selected astrocytes as the potential source of an ECM. Astrocytes are cells responsible for flow regulation, energy metabolism, ion and water homeostasis, immune defense, neurotransmission, and adult neurogenesis [34]. We hypothesized that an astrocyte-derived ECM may have sufficient architectural and biochemical cues to promote the proliferation of NSCs. Using ECM on TCPS surfaces has been a successful strategy for improving the in vitro culture of several cell types including human chondrocytes, human adult mesenchymal stem cells, and cardiomyocytes [19,20,21].

Cell culture surfaces coated with the ECM deposited by human astrocytes were characterized in this work. We compared the attachment, proliferation rate, and modulation of the i-HNSC phenotype on an HAc with the biological responses of these cells on TCPS covered with an HiPSCc and a PLOc. The control surfaces were selected since these two coatings have been previously used for the in vitro culture and expansion of NSCs [35,36]. We confirmed the successful decellularization of the astrocyte monolayer using phase contrast microscopy and the even distribution of a rich layer of ECM components using SEM. Images of the surface at the micro-scale revealed significant differences in the appearance among the different coated surfaces. The HAc was a continuous structure and relatively smooth, while the HiPSCc was characterized by discrete pockets of ECM, and an absence of microstructures was observed in the PLOc. The differences in the coatings across the different experimental groups could be due to differences in the total amount of ECM deposited on the surface and the size of the molecules that were able to bind to TCPS. The HAc achieved saturation of the surface with astrocyte-derived ECM for several days, which resulted in the complete coverage of the surface, while the use of a coating solution for the HiPSCc limited the amount and identity of the ECM components that were able to bind to the surface. The HiPSCc was fabricated using the conditions and instructions provided by Cell Applications. The total concentration and identity of the ECM components in the HiPSCc solution is proprietary information and was not revealed by the manufacturer. PLO, on the other hand, is a single macromolecule with an average molecular weight between 70 kDa and 150 kDa. For the PLOc, the surface density of PLO was selected to fall in the middle range of coating levels of PLO (10 μg/mL–50 μg/mL) previously reported in the literature for the growth of NSCs [37,38]. It is not surprising that under the described experimental conditions, the characterization of the PLO-coated surface by SEM was not able to capture significant microstructures for this type of coating. In terms of the composition of the HAc, mass spectroscopy results agree with previously reported data in the literature. ECM components deposited by astrocytes are fibrillar proteins such as collagens; glycoproteins like laminins, fibronectin, and tenascins; as well as several classes of proteoglycans, specifically heparan sulfate, chondroitin sulfate, dermatan sulfate, and keratan sulfate proteoglycans [39,40,41]. In addition, growth factors and cytokines such as TGFB1 have also been reported as biomolecules normally secreted by astrocytes [42,43]. We believe that the deposition of these sets of macromolecules on the TCPS impacts the biological response of i-HNSCs. Several ECM components are known to play important roles in the NSC niche in the subventricular zone and subgranular zone, including tenascin, heparan sulfate, collagen IV, β1 integrin, and nidogen, all of which were identified in the astrocyte ECM, suggesting that this ECM contains necessary components to support the proliferation and self-renewal of NSCs. Here, the ECM may also be acting dynamically, as that of the neural fractones in the NSC niche in vivo, sequestering soluble biofactors and modulating their release.

Cell attachment 24 h after seeding on the HAc appears to be lower than on the other surfaces, although differences in attachment were not statistically significant. Although all surfaces were initially seeded using the same cell density, the extrapolated value for *X*_0_ from the exponential fitting suggests that relative to the HiPSCc, a lower fraction of the cells attached to the HAc during the initial lag phase of the growth process, which we estimated to be approximately 24 h. However, differences in the calculated *X*_0_ must be interpreted carefully due to the limited number of data points used for the exponential model. It is plausible that the differences in *X*_0_ between the HAc and the HiPSCc may have been obtained due to the lower average cell density in the HAc group at 24 h. In addition to the initial cell attachment, the proliferation rate of i-HNSCs on the selected surfaces was also estimated. Previously reported data for human adipose-derived and bone marrow-derived stem cells show that the doubling time is a function of the cell source and passage number. Chen et al. reported that the doubling time for human adult stem cells ranges from approximately 70 h to 200 h [44]. Similarly, Haragopal et al. estimated that the doubling time for HNSCs was approximately 150 h [45]. Our results for the doubling time of i-HNSCs (78.8 h and 49.9 h) on the HiPSCc and HAc, respectively, were within the previously reported range for human stem cells. The use of the HAc seems to reduce the doubling time of the cells to 49.9 h. However, this apparent increase in proliferation rate may be due to a mathematical artifact derived from the limited number of data points in the exponential model and the apparent lower initial cell density in the HAc group. Moreover, our HAc and the HiPSCc supported similar cell densities for the different time points and led to achieving similar cell number yields. In summary, our attachment and proliferation studies indicate that our proposed HAc is at least as good as the commercially available HiPSCc at supporting the attachment and the in vitro proliferation of i-HNSCs. The doubling time for the i-HNSCs on the PLOc could not be calculated. NSCs in this surface did not reach the exponential growth phase during the time frame of the experiment. While the cell density increased with time, the growth rate was very low.

In terms of phenotype modulation during NSC expansion, we investigated the regulation in the expression of two commonly used NSC stemness markers Sox2 and Nestin [46,47,48], and the early differentiation marker DCX at the gene and protein expression levels [49,50,51]. In the HAc, the gene expression of Sox2 and Nestin was significantly higher or at the same levels relative to the i-HNSCs and PLOc, respectively, and no significant differences were observed in the expression of DCX. Since significant differences were observed among experimental groups at the gene expression level, further examination of the regulation of these two stemness markers was performed at the protein expression level using immunostaining. Semiquantitative results indicate that the proposed HAc has the capacity to retain the stemness characteristics of NSCs at comparable levels observed in two commonly used coatings for the in vitro culture of NSCs. Overall, the HAc is a novel approach that supports the proliferation rate of NSCs in vitro while retaining their stemness. In addition, we also verified that i-HNSCs on our HAc retained the capacity to differentiate into neuron-like cells. The expression of Sox2 was below the detection limit for cells grown with NDM for 7 days. In addition, the expression of the immature neuron marker NeuroD1 and the mature markers RBFox3 and MAP2 were upregulated after 7 days in culture. As expected, the expression of the stemness marker was downregulated as the cells started differentiating into neuron-like cells, while the expression of neuron markers was enhanced. These results are very promising and could aid our research and the medical community in developing new and effective strategies for the establishment of in vitro NSC cultures that can rapidly yield therapeutic cell numbers while retaining their neural differentiation potential.

## 5. Conclusions

In this research work, we used an astrocyte-derived coating as a potential substrate for the in vitro culture of human neural stem cells. Astrocytes, as the main glial cell type in the CNS, are responsible for maintaining and supporting normal neuronal function. This work characterized the main components of astrocyte ECM deposited on a TCPS surface and retained after a detergent-based treatment. Mass spectroscopy revealed that the most abundant proteins in the HAc were fibronectin, heparan sulfate, tenascin, TGFB1, and serpin H1. Collagen I, collagen VI, collagen II, and collagen VIII were also present in the HAc. The HAc supported the attachment, proliferation, and overall cell yield to similar levels to those exhibited on the HiPSCc group. Improved proliferation and cell yield on the HAc were observed relative to the PLOc. Furthermore, i-HNSCs on the HAc retained the expression of the stemness markers Sox2 and Nestin at the gene and protein expression levels. Finally, the increase in the expression of the neural differentiation markers NeuroD1, RBFox3, and MAP2 confirmed that i-HNSCs cultured on the HAc using neural differentiation media retained their neural differentiation potential.

## Figures and Tables

**Table 1:** Primer sequences.

Gene	Sequence	Manufacturer
β-actin	F: CACCATTGGCAATGAGCGGTTC	Fisher-Eurofins
	R: AGGTCTTTGCGGATGTCCACGT	Fisher-Eurofins
Sox2	Proprietary sequence	Qiagen
Nestin	F: TCAAGATGTCCCTCAGCCTGGA	Fisher-Eurofins
	R: AAGCTGAGGGAAGTCTTGGAGC	Fisher-Eurofins
DCX	Proprietary sequence	Qiagen
NeuroD1	Proprietary sequence	Qiagen
RBFox3	Proprietary sequence	Qiagen
MAP2	F: AGGCTGTAGCAGTCCTGAAAGG	Fisher-Eurofins
	R: CTTCCTCCACTGTGACAGTCTG	Fisher-Eurofins

**Table 2:** Primary antibody information manufactured by Santa Cruz Biotechnology.

Marker Type	Primary Antibody	Clone	Catalog Number
Stemness	Sox2	E-4	sc-365823
Stemness	Nestin	10c2	sc-23927

## Data Availability

Data are contained within the article.

## Supplementary Materials

The following supporting information can be downloaded at: https://www.mdpi.com/article/10.3390/biomimetics8080589/s1, Figure S1: Representative fluorescence image of Sox2 staining without DAPI. Sox2 is present in both the nucleus and cytoplasm of the cells; scale bar = 100 μm.
