# Supplementary material for: A Bioinspired Astrocyte-Derived Coating Promotes the In Vitro Proliferation of Human Neural Stem Cells While Maintaining Their Stemness
Source: Biomimetics (Basel). 2023 Dec 4;8(8):589. doi: 10.3390/biomimetics8080589 (PMC10741944; doi:10.3390/biomimetics8080589)
Supplement: Supplementary file 1 [file biomimetics-08-00589-s001.zip › biomimetics-2681705-supplementary.pdf]

Supplementary Figure

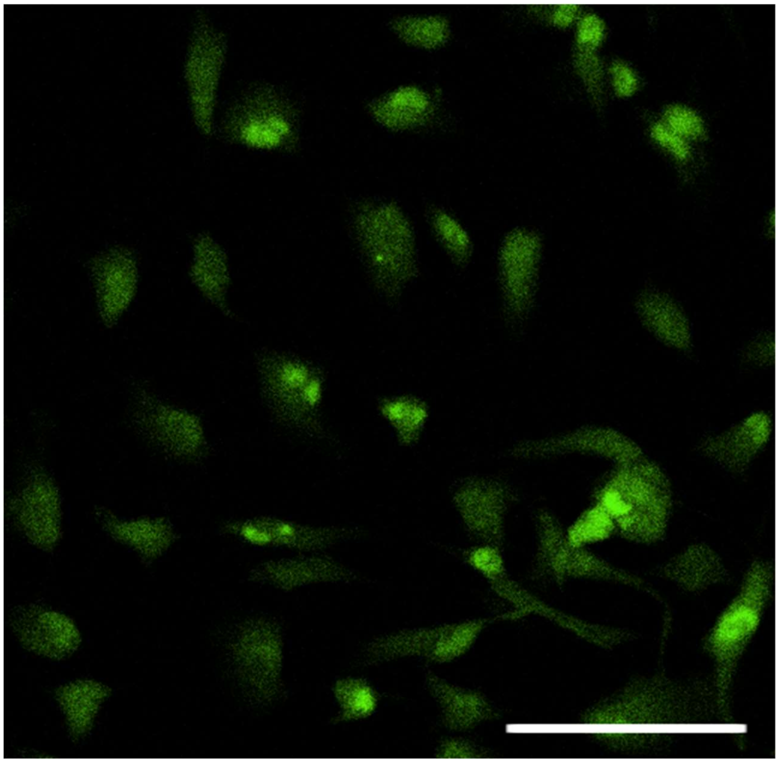

Figure S1. Representative fluorescence image of Sox2 staining without DAPI. Sox2 is present in both the nucleus and cytoplasm of the cells; scale bar = 100 μm.
